# Construction Notes

**Published:** 1893-05-27

**Authors:** 


### CONSTRUCTION NOTES.

St. George's Hospital.—The improvements effected at St. George's Hospital last year have proved most satisfactory. The re-arrangements of bath-rooms and lavatories attached to the wards were carried out by Messrs. Higgs and Hill, contractors.

The City of London Truss Society.—Improvements have been effected in the premises of this society, and £3,000 has been expended. The patients' waiting-rooms are excellently arranged, and are large, light and airy, and well-ventilated; while in winter time the whole will be heated by hot-air pipes; the seats are broad and comfortable, with sloping backs. The women's room will afford comfortable accommodation for 50, the men's room for a still larger number.

The foundation-stone of the Shaw Convalescent Home at Bearsden (Glasgow) was laid on the 9th ult. This institution is being erected by Miss Shaw in memory of the late brother, Mr. A. S. Shaw. To provide for its erection, equipment, and endowment, Miss Shaw set aside a sum of £40,000. The site secured for the Home is an admirable one. The Home is intended to accommodate 50 patients at a time, and when completed will be gifted to the Royal Infirmary. The main front building is to be three storeys in height with a basement, the whole having a frontage of 176 feet, and being treated in the modern Gothic style. The centre of the structure will be adorned with a lofty square tower. From the main entrance the principal corridors lead right and left under the tower to the day rooms on the ground floor, the staircase leading to the dormitories in the upper flats. The dining hall has separate entrances for male and female patients, and has immediate service from the kitchen adjoining. Besides a recreation-room there will be a smoking room for the men and a day working-room for the women, and there is ample provision for nurses, as well as lavatory, bath-room, and cloak-room accommodation. The grounds extend in all to about 12 acres, to be laid out for recreation purposes. The contractors for the work are Messrs. George Barlas and Co., of Hillhead, and the buildings are erected from plans prepared by Mr. James Thomson, F.R.I.B.A., of Bath Street, Glasgow.

